# The Impact of Posterior Maxillary Teeth on Maxillary Sinus: Insights From Cone-Beam Computed Tomography Analysis

**DOI:** 10.7759/cureus.76578

**Published:** 2024-12-29

**Authors:** Marwa Khalil, Dina Attia, Haitham H Sakr

**Affiliations:** 1 Oral Medicine, Periodontology, Oral Diagnosis, and Oral Radiology, Alexandria University, Faculty of Dentistry, Alexandria, EGY; 2 Pediatric Dentistry and Dental Public Health, Alexandria University, Faculty of Dentistry, Alexandria, EGY; 3 Faculty of Dentistry, Pharos University, Alexandria, EGY

**Keywords:** cone- beam computed tomography, maxillary sinus mucosal thickening, maxillary teeth, periapical lesions, periodontal disease (pd)

## Abstract

Background

Odontogenic maxillary sinusitis arises mainly from dental origins, emphasizing the connection between dental health and sinus issues. Understanding these relationships is crucial for implant planning, sinus augmentation procedures, and managing post-extraction complications. This knowledge can help clinicians make informed decisions about treatment timing and approach. In this study, the influence of tooth periapical and periodontal conditions on maxillary sinus mucous membrane thickening using cone-beam computed tomography (CBCT) imaging was evaluated.

Methods

In this retrospective cross-sectional study, 124 patients who underwent CBCT for dental treatment covering 248 maxillary sinuses and 992 teeth were examined. Coronal and sagittal sections were examined to assess the maxillary sinus mucosal thickening (MT), the periodontal bone loss for maxillary posterior teeth, and their proximity to the maxillary sinus (RPMS). The mean ± standard deviation of quantitative variables was compared using a one-way ANOVA test. Multivariable logistic regression analysis was used to assess the relation between the different independent variables and MT of the maxillary sinus. P < 0.05 was considered to be statistically significant.

Results

MT was detected in 90 (72.6%) patients. We found 42 (33.9%) patients bilaterally. Grade 1 mucosal thickness was the most prevalent grade (72.5%), followed by Grade 2 (19.2%). Almost half the teeth examined were not in contact with the cortical borders of the sinus (56.0%). Most of the teeth showed mild to moderate periodontal bone loss (80.2%). Logistic regression analysis showed that the variables significantly associated with higher odds of MT of maxillary sinus occurrence were tooth location (second molar vs. first premolar AOR = 2.30, 95% CI: 1.29-2.79, first molar vs. first premolar AOR = 3.79, 95% CI: 1.82- 3.10, and second premolar vs. first premolar AOR = 1.57, 95% CI: 1.27-2.84), the relation of roots of posterior maxillary teeth to the sinus floor (Type 2 vs. Type 0 AOR = 2.27, 95% CI: 1.38-3.34, and Type 1 vs. Type 0 AOR = 2.24, 95% CI: 1.54-3.26), periodontal bone loss (severe vs. non-periodontitis AOR = 2.75, 95%CI: 1.29-5.86, and mild-moderate periodontitis vs. non-periodontitis AOR = 1.68, 95%CI: 1.19-4.93), and periapical and periodontal tooth condition (periapical lesion vs. extracted AOR = 32.7, 95% CI: 4.53-83.61, and no periapical lesion vs. extracted AOR = 19.8, 95% CI: 8.18-74.23).

Conclusions

Second molars, severe periodontal bone loss, and periapical lesions were associated with increased sinus MT risk, highlighting the need for dental health consideration in maxillary sinus management.

## Introduction

The maxillary sinus (MS), an air-filled paranasal cavity within the maxilla, plays a significant role in both dental health and pathology. Its inner lining, the Schneiderian membrane (SM), is a highly vascularized layer of pseudostratified ciliated epithelium, typically 0.8-1.0 mm thick [1,2]. Sinus membrane thickening (SMT), a prevalent MS abnormality, arises from an inflammatory reaction often spurred by infection or allergic stimulation, which can augment SM thickness dramatically [3]. This condition, defined as mucosal thickness exceeding 2 or 3 mm, is commonly odontogenic in origin. This condition affects 10-51.8% of dental patients [4], impacting treatment planning for implants, extractions, and periodontal procedures.

Odontogenic maxillary sinusitis (OMS), inflammation triggered primarily by dental sources, emphasizes the intricate relationship between dental health and sinus pathology [4]. Conditions such as periodontitis, apical pathology, and dental treatments have been identified as leading contributors to OMS, highlighting the impact of dental health on sinus conditions [5]. Furthermore, the extraction of posterior maxillary teeth can induce alveolar ridge remodeling and maxillary sinus pneumatization (MSP), affecting future implant placements and altering the mechanical strength of adjacent bone tissues [6]. Previous studies indicated that 30-40% of dental patients exhibit some degree of sinus MT, necessitating modifications in treatment approaches, particularly for implant placement and periodontal procedures [3-5].

The periapical infection has been shown to affect the sinus mucosa even without perforation of the cortical sinus floor, with the infection spreading via bone marrow, blood vessels, and lymphatics to the sinus. Bacterial toxins and products of pulpal necrosis may spread to the MS and lead to inflammation [7]. Sinus MT can affect systemic health through chronic inflammation and increased risk of sinusitis, potentially complicating dental procedures and impacting overall patient well-being [8,9].

In this context, cone-beam computed tomography (CBCT) emerges as an invaluable tool for diagnosing and understanding the complex anatomical and pathological interactions between the maxillary sinus and posterior maxillary dentition [10]. Offering superior resolution and three-dimensional visualization, CBCT aids in the detailed assessment of odontogenic etiologies and their impact on MS, providing a foundation for targeted interventions and treatment strategies [10].

Moreover, the role of periodontitis as a precursor to sinus mucosal thickening has been increasingly recognized. Multiple studies [11-13] have demonstrated that the inflammatory cascade initiated by periodontal disease is known to extend beyond the confines of the oral cavity, potentially affecting the maxillary sinus and leading to mucosal thickening. Maska et al. [13] highlight the necessity of considering periodontal health in patients undergoing sinus floor elevation and implant placement, as the presence of periodontal disease was significantly associated with mucosal thickening in the maxillary sinus. Thus, the objective of this retrospective cross-sectional study was to evaluate the relationship between maxillary sinus mucosal thickening and three dental factors: the location of posterior teeth (first/second premolars and molars); their periodontal status; and the proximity of their roots to the sinus floor using CBCT imaging.

## Materials and methods

This study is a retrospective cross-sectional analysis that utilized cone-beam computed tomography (CBCT) images of patients who were seeking dental treatment at the Faculty of Dentistry, Pharos University, from February 2023 to February 2024. The research received ethical approval from the Pharos University Institutional Review Board (PH, IRB-3-058) and was conducted according to the Strengthening the Reporting of Observational Studies in Epidemiology (STROBE) standards [14]. This study ensured all participants provided written consent for their CBCT images to be used for research, including potential future studies. This consent process guaranteed the anonymity and confidentiality of patient data, adhering to ethical guidelines.

Participants included in this study were non-smoking adult patients of any gender, aged from 25 to 55 years, seeking dental care for various procedures, including tooth extractions, endodontic or prosthodontic treatments, as well as implant planning. Exclusion criteria encompassed individuals younger than 25 years, patients with systemic conditions or on medication affecting bone metabolism or skeletal anomalies, and partial CBCT scans or CBCT scans with artifacts were excluded. Also, patients with incomplete demographic data or medical history or having bone diseases, such as osteoporosis, Paget's disease, and bone metabolic disorders, were excluded from the study.

Cone-beam computed tomography imaging technique 

The CBCT imaging was performed utilizing the Morita 3D units (J. MORITA CORP, Japan), featuring a flat panel detector. The imaging area was defined as a cylinder with dimensions ranging from 15 to 20.6 cm in height and 9 to 18 cm in diameter, captured at a standard resolution with a voxel size of 0.2 mm. The device settings were adjusted to a tube voltage of 90 kV, a tube current of 10 mA, and an exposure duration of 10.8 seconds. Analysis of the DICOM files from the CBCT scans was conducted using i-Dixel 2.0 imaging software (J. MORITA CORP, Japan), with a comprehensive review of the full volume data.

Classification of root tip proximity and sinus floor configuration 

**Figure 1:**
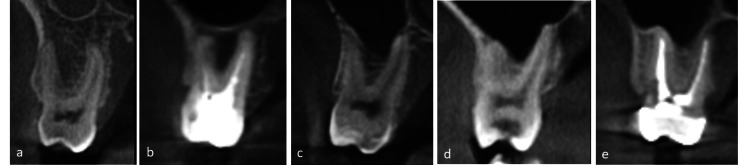
Cone-beam computed tomography Coronal sections showing proximity of the root tips of posterior maxillary teeth (RPMT) to the sinus floor: a) Type 0: no contact with the sinus cortical borders; b) Type 1: the root tips are in contact with the cortical borders of the sinus with an inferiorly curving sinus floor; c) Type 2: the root tips are projecting laterally on the sinus cavity, but the apex is outside the sinus boundaries with an inferiorly curving sinus floor; d) Type 3: the root tips are projecting into the sinus cavity with an inferiorly curving sinus floor; e) Type 4: a superiorly curving sinus floor enveloping part or all of the root tips.

**Figure 2:**
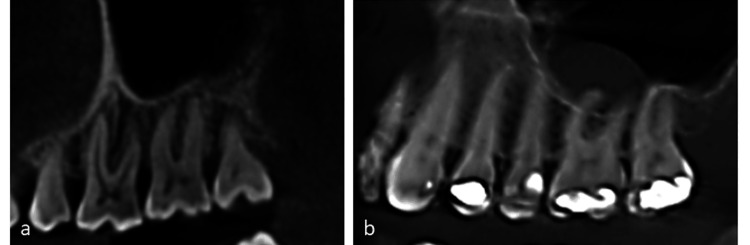
Cone-beam computed tomography sagittal sections a) no contact with root tips to the sinus cortical borders; b) the root tips are projecting into the sinus cavity.

The root tips proximity to the sinus floor (RPMT) was evaluated on the coronal and sagittal CBCT sections for maxillary premolars and first and second molars according to Sharan et al. [15] (Figures 1, 2): Type 0: no contact between root apex and the sinus cortical borders; Type 1: root apex is in contact with the sinus cortical borders and with an inferiorly curving floor; Type 2: root apex is laterally adjacent to the sinus cavity, but the apex is outside the sinus; Type 3: root apex protruding into the sinus with an inferiorly curving floor; Type 4: the sinus floor enclosing part or all of the root apex.

Maxillary sinus mucosal thickening and periodontal bone loss assessment

**Figure 3:**
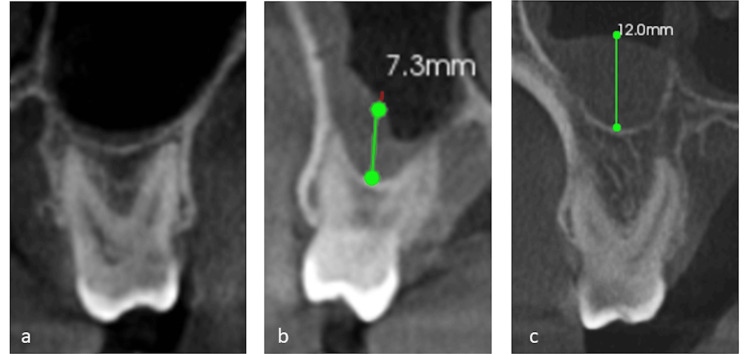
Coronal sections of cone-beam computed tomography images showing mucosal thickening grades a) Grade 1: 0-2 mm; b) Grade 2: 2-10 mm; and c) Grade 3: more than 10 mm.

Mucosal thickening within the maxillary sinus was assessed on coronal CBCT sections at the point of greatest thickness from the sinus floor, categorized into three grades [16] (Figure 3): Grade 1 denotes 0 to 2 mm (normal), Grade 2 spans 2 to 10 mm (moderate), and Grade 3 exceeds 10 mm (severe) thickening. Radiographic bone loss was determined 2 mm apical from the cement-enamel junction (CEJ) to the alveolar crest, and the percentage of bone loss per tooth was calculated by measuring from the CEJ to the root tip, adjusting for alveolar crest height, and then standardizing the measurement across all teeth to determine overall bone loss. The periodontal bone loss for all maxillary posterior teeth was then classified according to Engebretson et al. [17] as Type 1 (normal to mild, < 25% bone loss), Type 2 (moderate, 25 to 50% bone loss), and Type 3 (severe, > 50% bone loss).

Reliability of data assessment 

To ensure the reliability of data, intra- and inter-examiner calibrations were conducted using 15 randomly chosen CBCT scans. Two examiners, previously calibrated, participated in a training session to align with the assessment criteria and protocol, followed by a comparative analysis to establish inter-examiner consistency. Measurements were repeated after 10 days to determine intra-examiner consistency. The agreement level was calculated using Cohen's kappa coefficient, with any discrepancies during CBCT evaluation being collaboratively resolved to reach a consensus. A third experienced radiologist, not involved in initial measurements, served as an independent evaluator. In case of disagreement between the two evaluators, the third independent reviewer's opinion was used to resolve the disagreement. Final measurements were determined through consensus meetings where all three evaluators reviewed disputed cases together, and the median of three measurements was used.

Statistical analysis

Statistical analysis was carried out using the statistical package for the social sciences IBM SPSS Statistics for Windows (Mac OS X, Apple Inc., California, United States), Version 28 (Released 2021; IBM Corp., Armonk, New York, United States). Descriptive statistics were displayed as the mean ± standard deviation for quantitative variables, while frequencies and percentages were used for qualitative variables. The study groups were compared regarding demographic factors and odontogenic factors using the chi-square test. The mean values of quantitative variables were compared using a one-way ANOVA test followed by a post hoc Tukey test. Multivariable logistic regression analysis was used to assess the relation between the different independent variables and the MT of maxillary sinus occurrence (present/absent). The reference categories in the multivariable logistic regression analysis were selected based on clinical relevance and interpretability. For tooth location, the first premolar was chosen as the reference because it typically exhibits the least proximity to the sinus floor. For the relation of roots of posterior maxillary teeth to the sinus floor (RPMT), Type 0 (no relation) was used as the baseline due to its lack of anatomical interaction with the sinus. Non-periodontitis (<25% bone loss) served as the reference for periodontal bone loss, as it represents the healthiest condition, and extracted teeth were the reference for tooth condition to provide a neutral baseline. P < 0.05 was considered to be statistically significant.

## Results

The current study enrolled 124 patients who underwent CBCT covering 248 maxillary sinuses (124 right and 124 left), and 992 teeth were examined. The intra- and inter-examiner agreement showed a κ-coefficient of 0.98 and 0.97, respectively. Statistical analysis was conducted using the average of both examiners' scores. Table 1 shows that the sample was equally distributed in terms of gender (50.0% males). The patient's ages ranged from 25 to 55 years; the mean ± SD age was 35 ± 4.4. Mucosal thickness was detected in 90 (72.6%) cases, 19 (15.3%) of which were found on the right side, 29 (23.4%) were found on the left side, and 42 (33.9%) were found bilaterally. Grade 1 mucosal thickness was the most prevalent grade (72.5%), followed by Grade 2 (19.2%).

**Table 1:** Sample characteristics (N = 124 individuals) MT: mucosal thickening

Sample details			n	%
Gender	Male		62	50.0
	Female		62	50.0
Prevalence of MT	Yes		90	72.6
	No		34	27.4
Distribution of MT	Unilateral	Right	19	15.3
		Left	29	23.4
	Bilateral		42	33.9
Mucosal thickness grading (N= 992 teeth)	Grade 1 (0-2 mm)		719	72.5
	Grade 2 (2-10 mm)		190	19.2
	Grade 3 (>10 mm)		83	8.4

The oral characteristics of examined teeth are shown in Table 2. Almost half the teeth examined were not in contact with the cortical borders of the sinus (56.0%). Most of the teeth showed mild to moderate periodontal bone loss (80.2%) and had no periapical lesion (82.1%). The first molar and second premolar teeth had higher average root lengths among the examined teeth (mean ± SD 11.8 ± 5.1, 11.8 ± 5.6, respectively). As for the average root lengths (mean ± SD), they were 11.6 ± 5.0 mm for second molars, 11.8 ± 5.1 mm for first molars, 11.8 ± 5.6 mm for second premolars, and 11.3 ± 5.4 mm for first premolars.

**Table 2:** Oral characteristics of teeth examined (N = 992 teeth)

Oral characteristics		n		%
The relation of roots of posterior maxillary teeth to the sinus floor (RPMT)	Type 0	556		56.0
	Type 1	278		28.0
	Type 2	117		11.8
	Type 3	36		3.6
	Type 4	5		0.5
Periodontitis condition	Non-periodontitis	160		16.1
	Mild-Moderate (25% - 50%)	796		80.2
	Advanced (>50 %)	36		3.7
Teeth condition	Missing	160		16.1
	No periapical lesion	814		82.1
	Periapical lesion	18		1.8

Table 3 shows that the MT of the maxillary sinus was significantly associated with tooth location (p < 0.001), the relation of roots of posterior maxillary teeth to the sinus floor (p < 0.001), periodontal bone loss (p < 0.001), teeth condition (p < 0.001), and root length teeth (p = 0.04), but not the gender of patients (p = 0.28).

**Table 3:** Comparison analysis of mucosal thickness of maxillary sinus according to demographic and odontogenic variables (N = 992 teeth) $ Test of significance: chi-squared test; † Test of significance: one-way ANOVA. There is no statistically significant difference between root lengths with same letters at 0.05 according to post hoc Tukey test. *Significant at P ≤ 0.05.

Variables		Mucosal thickness			P value^$^
		Grade 1, n (%)	Grade 2, n (%)	Grade 3, n (%)	
Gender^$^	Male	361 (72.8)	100 (20.2)	35 (7.1)	0.28
	Female	358 (72.2)	90 (18.1)	48 (9.7)	
Tooth location^$^	2^nd^ molar	176 (71.0)	53 (21.4)	19 (7.7)	< 0.001*
	1^st^ molar	146 (58.9)	69 (27.8)	33 (13.3)	
	2^nd^ premolar	187 (75.4)	40 (16.1)	21 (8.5)	
	1^st^ premolar	210 (84.7)	28 (11.3)	10 (4.0)	
The relation of roots of posterior maxillary teeth to the sinus floor^$ ^(RPMT)	Type 0	421 (72.2)	114 (19.6)	48 (8.2)	< 0.001*
	Type 1	216 (53.6)	119 (29.5)	68 (16.9)	
	Type 2	87 (47.5)	57 (31.1)	39 (21.3)	
	Type 3	39 (50.6)	19 (24.7)	19 (24.7)	
	Type 4	7 (30.4)	4 (17.4)	12 (52.2)	
Periodontal bone loss^$^	Non-periodontitis <25%	155 (96.9)	3 (1.9)	2 (1.3)	< 0.001*
	Mild-moderate 25% - 50%	54 (68.1)	172 (21.6)	82 (10.3)	
	Severe >50%	19 (52.8)	14 (38.9)	3 (8.3)	
Tooth condition^$^	Extracted	158 (98.8)	0 (0)	2 (1.3)	< 0.001*
	No periapical lesion	551 (67.7)	183 (22.5)	80 (9.8)	
	Periapical lesion	10 (55.6)	7 (38.9)	1 (5.6)	
Root length^† ^(mean ± SD)	2nd molar	10.9 ± 5.7^(a)^	13.2 ± 2.1^(b)^	13.6 ± 1.5^(a,b)^	0.04*
	1st molar	10.4 ± 6.0^(a)^	13.8 ± 1.8^(b)^	13.4 ± 1.3^(c)^	
	2nd premolar	11.2 ± 6.0^(a)^	14.4 ± 3.1^(b)^	12.5 ± 4.4^(b)^	
	1st premolar	10.8 ± 5.7^(a)^	13.7 ± 2.2^(b)^	14.4 ± 0.68^(a,b)^	

The results of logistic regression analysis are shown in Table 4. The variables significantly associated with higher odds of MT of maxillary sinus occurrence were tooth location (second molar vs. first premolar AOR = 2.30, 95% CI: 1.29-2.79, first molar vs. first premolar AOR = 3.79, 95% CI: 1.82- 3.10, and second premolar vs. first premolar AOR = 1.57, 95% CI: 1.27-2.84), the relation of roots of posterior maxillary teeth to the sinus floor (Type 2 vs. Type 0 AOR= 2.27, 95% CI: 1.38-3.34, and Type 1 vs. Type 0 AOR = 2.24, 95% CI: 1.54-3.26), periodontal bone loss (severe vs. non-periodontitis AOR = 2.75, 95%CI: 1.29-5.86, and mild-moderate periodontitis vs. non-periodontitis AOR = 1.68, 95%CI: 1.19-4.93), and tooth condition (periapical lesion vs. extracted AOR = 32.7, 95% CI: 4.53-83.61, and no periapical lesion vs. extracted AOR = 19.8, 95% CI: 8.18-74.23).

**Table 4:** Association between odontogenic factors and MT of maxillary sinus occurrence in multivariable logistic regression MT: mucosal thickening; AOR: adjusted odds ratio; CI: confidence interval; *significant at P ≤ 0.05.

Factors		Mucosal thickness of maxillary sinus presence			
		AOR	95% CI		P value
Tooth location	2^nd^ molar	2.30	1.29, 2.79		< 0.001*
	1^st^ molar	3.97	1.82, 3.10		< 0.001*
	2^nd^ premolar	1.57	1.27, 2.84		0.03*
	1^st^ premolar	Reference			
The relation of roots of posterior maxillary teeth to the sinus floor (RPMT)	Type 4	2.52	0.40, 15.83		0.33
	Type 3	1.74	0.80, 3.77		0.16
	Type 2	2.27	1.38, 3.34		0.001*
	Type 1	2.24	1.54, 3.26		< 0.001*
	Type 0	Reference			
Periodontal bone loss	Severe >50%	2.75	1.29, 5.86		0.009*
	Mild-moderate 25% - 50%	1.68	1.19, 4.93		0.03*
	Non-periodontitis <25%	Reference			
Tooth condition	Periapical lesion	32.7	4.53, 83.61	<0.001*	
	No Periapical lesion	19.8	8.18, 74.23	<0.001*	
	Extracted	Reference			
Root length		0.94	0.86, 1.13	0.68	

## Discussion

The study investigated the impact of various factors on maxillary sinus mucosal thickening (MT) using CBCT imaging across 124 individuals, analyzing 992 teeth, with an equal gender distribution (50% male, 50% female) in MT. Previous studies suggested that odontogenic factors, notably severe periodontitis and chronic periapical periodontitis, account for up to 40% of maxillary sinus inflammation cases. These dental issues frequently accompany other oral health challenges, such as missing teeth, alveolar bone defects, and maxillary sinus mucosal thickening. These conditions present significant obstacles to implant placement following teeth extraction [18,19].

Huang et al. [20] showed in their comprehensive review that the cut-off thickness to distinguish between normal mucosa and a sick condition by CBCT scan image was 2 mm. Therefore, a sinus membrane was considered pathogenic if the mucosa thickened by more than 2 mm. They concluded that the sinus membrane thickness was significantly and positively linked with periodontal bone loss and periapical diseases. The current findings showed that there was a significant association between MT and both periodontal bone loss and periapical lesions, emphasizing the influence of dental health on sinus conditions. Song et al. [21] evaluated the CBCT of 93 patients with maxillary posterior periapical periodontitis or periodontitis. They observed that periodontitis patients had significantly thicker preoperative maxillary sinus mucosa than those with periapical periodontitis. Statistical analysis showed significant variations in mucosal thickness changes over time and with inflammation severity. The study suggests extracting infected teeth and thorough debridement for patients with sinus mucosa thickening due to these conditions, recommending maxillary sinus augmentation three to six months post-extraction for optimal outcomes. However, other factors may contribute to MT even in the absence of odontogenic causes, such as allergic rhinitis, chronic sinusitis, or incidental mild thickening observed in asymptomatic individuals [19].

The results showed a significant association between maxillary sinus MT with teeth condition and root length. Althobiti et al. [22] conducted a retrospective analysis on 364 individuals, focusing on the relationship between maxillary sinus mucosal (MSM) thickness, periapical lesions, and other factors using CBCT images. Findings revealed a significant correlation between MSM thickness and periapical index (PAI), particularly noting a marked increase in MSM thickness with higher PAI scores. While age showed no significant correlation with MSM thickening, gender did, suggesting a clear link between periapical lesions and MSM thickening.

The majority of teeth (72.5%) were associated with Grade 1 mucosal thickness (0-2 mm), indicating that mild mucosal thickening is the most common finding. However, the presence of Grade 2 (19.2%) and Grade 3 (8.4%) thickening suggests a considerable number of individuals experience significant sinus mucosal changes. These gradings can be essential for correlating dental conditions with the severity of sinus mucosal responses. Previous CBCT studies [23,24] reported a similar prevalence of mucosal thickening that ranged from 24% (19) up to 38% [24].

Huang et al. [20] investigated the relationship between the health of maxillary posterior teeth and maxillary sinus membrane thickening using cone-beam computed tomography (CBCT) images from 235 Taiwanese patients. The research identified a significant prevalence of maxillary sinus membrane thickening, defined as >2 mm, observed in 36.6% of the cases. Notably, the study found strong associations between sinus membrane thickening and dental health issues such as periodontal bone loss and periapical lesions, both showing statistical significance (p < 0.001). Specifically, severe periodontal and mild-moderate bone loss elevated the risk of sinus membrane thickening, with odds ratios of 2.75 and 1.68, respectively. These findings highlighted the importance of considering dental health conditions and demographic factors when assessing the risk for maxillary sinus membrane thickening.

The current findings showed that second molars had a higher odds ratio for MT, highlighting the role of tooth position and condition in sinus health. Almost half of the teeth examined (46%) showed that the relation of the roots to the sinus floor ranged from Type 1 to 4. This suggests that a significant portion of the posterior maxillary teeth have directly influenced sinus conditions through root proximity. However, the presence of Types 1 through 4, especially Type 1 (28%) and Type 2 (11.8%), indicates a considerable number of teeth have roots extending into or very close to the sinus floor, which could potentially affect the maxillary sinus mucosa.

Previous studies [25,26] observed that RPMT Type 0 was most frequently observed in premolars and Type 3 in molars. Oberli et al. [26] found after examining 113 radiographs that 70.4% of patients had a space between the root tip and the sinus floor, and in almost 11% of cases, the root tip made contact with the sinus floor. To determine where the posterior roots are in relation to the maxillary sinus floor, previous radiographic examinations have used panoramic radiographs [25]. However, the ability of panoramic radiography to predict the root position and root projection of posterior maxillary teeth in relation to the maxillary sinus was disputed [27]. Therefore, CBCT and panoramic radiography were compared by Jung and Cho [27] for the evaluation of radiographic indications indicating root extension into the maxillary sinus, showing that panoramic radiography was reliable when the root was not in contact with the sinus floor. On panoramic radiographs, however, the root extending into the sinus exhibited a modest capacity to predict root protrusion into the maxillary sinus.

While we acknowledge that the selected age range excludes older populations with higher periodontal disease prevalence, this limitation allows for a more controlled analysis of the direct relationship between dental conditions and sinus mucosal thickening, minimizing age-related variables that could confound results. This range represents the primary demographic seeking elective dental procedures and implant therapy, allowing findings to be most applicable to routine clinical practice. Future studies specifically examining older populations would complement these findings.

 In the current study, while gender distribution was equal, the lack of significant gender differences suggests that sinus MT may be primarily influenced by local anatomical and pathological factors rather than gender-specific characteristics.

The severity of maxillary sinus MT has been linked to the extent of apical periodontitis [28]. This has been attributed to the infiltration of pathogenic bacteria, toxins, and inflammatory cytokines from apical lesions, either directly through the porous maxillary bone or indirectly via the bloodstream and lymphatic system [28]. Consequently, an increase in these harmful agents can exacerbate periapical lesions, elevating the risk of MT in the maxillary sinus [4,28]. These findings suggest implementing pre-treatment CBCT screening for high-risk cases, especially for second molars with periodontal involvement. Collaboration between dental specialists and otolaryngologists is recommended for cases with significant MT. Although periapical lesions are a recognized cause of MT, other factors may contribute to MT even in their absence. For instance, periodontal bone loss can lead to inflammatory changes in the sinus mucosa due to the anatomical proximity of posterior teeth to the sinus. Additionally, non-odontogenic causes such as allergic rhinitis or chronic sinusitis may independently induce mucosal thickening. Physiological variations, such as incidental mild thickening observed in asymptomatic individuals, may also account for these findings [19].

The prevention and management of sinus mucosal thickening necessitates timely periodontal intervention [11]. Early detection and treatment of periodontal disease through scaling and root planing, combined with regular maintenance protocols, can significantly reduce the risk of sinus complications [4]. In cases with established periodontal involvement, a comprehensive treatment approach along with surgical intervention for severe cases should be implemented before significant bone loss occurs [29]. This preventive approach is particularly crucial for teeth adjacent to the maxillary sinus, where periodontal health maintenance plays a vital role in preventing sinus pathology [29] and reducing potential complications during future dental procedures, especially implant placement [30]. 

While the study provides valuable insights, the study's limitations are the retrospective design, which may not fully capture longitudinal changes or causality. The reliance on CBCT imaging, although precise, might not account for all variables affecting sinus health. Further, the study's demographic scope, primarily within a specific age range, may not generalize across all populations.

## Conclusions

The study reveals that mucosal thickening in the maxillary sinus is significantly influenced by tooth location, its proximity to the sinus floor, periapical and periodontal conditions, and tooth condition, with no significant gender differences. Higher odds of mucosal thickening were particularly noted with second molars, severe periodontal bone loss, and periapical lesions. These findings highlight the importance of an interdisciplinary approach involving dentists, periodontists, and otolaryngologists, particularly in cases with significant mucosal thickening or complex periodontal conditions. Regular monitoring of at-risk teeth, especially second molars with periodontal involvement, and early intervention strategies should be implemented to prevent or minimize sinus MT development. This collaborative approach, combined with thorough radiographic assessment using CBCT, can enhance treatment planning and improve long-term success rates in dental procedures involving the maxillary sinus region.

## References

[1] Van Den Munckhof T, Patel S, Koller G, Berkhout E, Mannocci F, Foschi F (2020). Schneiderian membrane thickness variation following endodontic procedures: a retrospective cone beam computed tomography study. BMC Oral Health.

[2] Lin YH, Yang YC, Wen SC, Wang HL (2016). The influence of sinus membrane thickness upon membrane perforation during lateral window sinus augmentation. Clin Oral Implants Res.

[3] Rege IC, Sousa TO, Leles CR, Mendonça EF (2012). Occurrence of maxillary sinus abnormalities detected by cone beam CT in asymptomatic patients. BMC Oral Health.

[4] Psillas G, Papaioannou D, Petsali S, Dimas GG, Constantinidis J (2021). Odontogenic maxillary sinusitis: a comprehensive review. J Dent Sci.

[5] Patel NA, Ferguson BJ (2012). Odontogenic sinusitis: an ancient but under-appreciated cause of maxillary sinusitis. Curr Opin Otolaryngol Head Neck Surg.

[6] Levi I, Halperin-Sternfeld M, Horwitz J, Zigdon-Giladi H, Machtei EE (2017). Dimensional changes of the maxillary sinus following tooth extraction in the posterior maxilla with and without socket preservation. Clin Implant Dent Relat Res.

[7] Obayashi N, Ariji Y, Goto M (2004). Spread of odontogenic infection originating in the maxillary teeth: computerized tomographic assessment. Oral Surg Oral Med Oral Pathol Oral Radiol Endod.

[8] Albert D, Clarkin C, Komoroski J, Brensinger CM, Berlin JA (2004). Wegener's granulomatosis: possible role of environmental agents in its pathogenesis. Arthritis Rheum.

[9] Kronzer VL, Davis JM, Hanson AC (2024). Association between sinusitis and incident rheumatic diseases: a population-based study. RMD Open.

[10] Yeung AW, Hung KF, Li DT, Leung YY (2022). The use of CBCT in evaluating the health and pathology of the maxillary sinus. Diagnostics (Basel).

[11] Ren S, Zhao H, Liu J, Wang Q, Pan Y (2015). Significance of maxillary sinus mucosal thickening in patients with periodontal disease. Int Dent J.

[12] Lathiya VN, Kolte AP, Kolte RA, Mody DR (2019). Analysis of association between periodontal disease and thickness of maxillary sinus mucosa using cone beam computed tomography - a retrospective study. Saudi Dent J.

[13] Maska B, Lin GH, Othman A, Behdin S, Travan S, Benavides E, Kapila Y (2017). Dental implants and grafting success remain high despite large variations in maxillary sinus mucosal thickening. Int J Implant Dent.

[14] von Elm E, Altman DG, Egger M, Pocock SJ, Gøtzsche PC, Vandenbroucke JP (2007). The Strengthening the Reporting of Observational Studies in Epidemiology (STROBE) statement: guidelines for reporting observational studies. Epidemiology.

[15] Sharan A, Madjar D (2008). Maxillary sinus pneumatization following extractions: a radiographic study. Int J Oral Maxillofac Implants.

[16] Cagici CA, Yilmazer C, Hurcan C, Ozer C, Ozer F (2009). Appropriate interslice gap for screening coronal paranasal sinus tomography for mucosal thickening. Eur Arch Otorhinolaryngol.

[17] Engebretson SP, Lamster IB, Elkind MS (2005). Radiographic measures of chronic periodontitis and carotid artery plaque. Stroke.

[18] Aukštakalnis R, Simonavičiūtė R, Simuntis R (2018). Treatment options for odontogenic maxillary sinusitis: a review. Stomatologija.

[19] Simuntis R, Tušas P, Kubilius R, Leketas M, Šiupšinskienė N, Vaitkus S (2020). Association between maxillary posterior teeth periapical odontogenic lesions and maxillary sinus mucosal thickening: a 3D volumetric computed tomography analysis. Sinusitis.

[20] Huang YT, Hu SW, Huang JY, Chang YC (2021). Assessment of relationship between maxillary sinus membrane thickening and the adjacent teeth health by cone-beam computed tomography. J Dent Sci.

[21] Song Y, Rong M, Ye Y (2024). Pathogenic factors of maxillary sinus mucosal thickening observed by cone-beam computed tomography (CBCT). Appl Radiat Isot.

[22] Althobiti GA, Alzaidi TA, Almingash JM, Alobaida RM, ALYahya RE, Binthunayyan SN (2024). Association between periapical odontogenic lesions and maxillary sinus mucosal thickening: a retrospective computed tomography analysis. Saudi Endod J.

[23] Pazera P, Bornstein MM, Pazera A, Sendi P, Katsaros C (2011). Incidental maxillary sinus findings in orthodontic patients: a radiographic analysis using cone-beam computed tomography (CBCT). Orthod Craniofac Res.

[24] Janner SF, Caversaccio MD, Dubach P, Sendi P, Buser D, Bornstein MM (2011). Characteristics and dimensions of the Schneiderian membrane: a radiographic analysis using cone beam computed tomography in patients referred for dental implant surgery in the posterior maxilla. Clin Oral Implants Res.

[25] Themkumkwun S, Kitisubkanchana J, Waikakul A, Boonsiriseth K (2019). Maxillary molar root protrusion into the maxillary sinus: a comparison of cone beam computed tomography and panoramic findings. Int J Oral Maxillofac Surg.

[26] Oberli K, Bornstein MM, von Arx T (2007). Periapical surgery and the maxillary sinus: radiographic parameters for clinical outcome. Oral Surg Oral Med Oral Pathol Oral Radiol Endod.

[27] Jung YH, Cho BH (2012). Assessment of the relationship between the maxillary molars and adjacent structures using cone beam computed tomography. Imaging Sci Dent.

[28] Zadsirjan S, Sheikhi M, Dakhilalian A, Feli M (2021). Association of inflammatory periapical lesions with maxillary sinus abnormalities: a retrospective cone-beam computed tomography study. J Dent (Shiraz).

[29] Ritter L, Lutz J, Neugebauer J (2011). Prevalence of pathologic findings in the maxillary sinus in cone-beam computerized tomography. Oral Surg Oral Med Oral Pathol Oral Radiol Endod.

[30] Wen SC, Lin YH, Yang YC, Wang HL (2015). The influence of sinus membrane thickness upon membrane perforation during transcrestal sinus lift procedure. Clin Oral Implants Res.

